# Effects of Purified Anthocyanins in People at Risk for Dementia: Study Protocol for a Phase II Randomized Controlled Trial

**DOI:** 10.3389/fneur.2020.00916

**Published:** 2020-09-02

**Authors:** Khadija Khalifa, Anne Katrine Bergland, Hogne Soennesyn, Ketil Oppedal, Ragnhild Oesterhus, Ingvild Dalen, Alf Inge Larsen, Tormod Fladby, Helen Brooker, Keith A. Wesnes, Clive Ballard, Dag Aarsland

**Affiliations:** ^1^Centre for Age-Related Medicine, Stavanger University Hospital, Stavanger, Norway; ^2^Department of Clinical Science, University of Bergen, Bergen, Norway; ^3^Stavanger Medical Imaging Laboratory (SMIL), Department of Radiology, Stavanger University Hospital, Stavanger, Norway; ^4^Department of Electrical Engineering and Computer Science, University of Stavanger, Stavanger, Norway; ^5^Department of Clinical Medicine, University of Bergen, Bergen, Norway; ^6^Section of Biostatistics, Department of Research, Stavanger University Hospital, Stavanger, Norway; ^7^Department of Cardiology, Stavanger University Hospital, Stavanger, Norway; ^8^Department of Neurology, Akershus University Hospital, Lørenskog, Norway; ^9^Institute of Clinical Medicine, University of Oslo, Oslo, Norway; ^10^Medical School, University of Exeter, Exeter, United Kingdom; ^11^Ecog Pro Ltd., Bristol, United Kingdom; ^12^Wesnes Cognition Ltd., Streatley, United Kingdom; ^13^Department of Psychology, Northumbria University, Newcastle, United Kingdom; ^14^Centre for Human Psychopharmacology, Swinburne University, Melbourne, VIC, Australia; ^15^Department of Old Age Psychiatry, King's College London, Institute of Psychiatry, Psychology and Neuroscience, London, United Kingdom

**Keywords:** anthocyanins, mild cognitive impairment, dementia, randomized controlled trial, intervention

## Abstract

**Background:** The number of people with dementia is increasing, with huge challenges for society and health-care systems. There are no disease-modifying therapies available. There is, therefore, an urgent need to identify strategies to reduce the risk of developing dementia. Anthocyanins are a class of compounds found in dark berries and fruits with some effects that might reduce the risk for cognitive decline and the development of dementia in older people.

**Aim:** This phase II three-center, randomized, 24-week, placebo-controlled study, ongoing in Norway, aims to evaluate the safety, and efficacy of anthocyanins in modifying key dementia-related mechanisms and maintain cognitive functioning in older people at risk for dementia.

**Methods:** Participants (220 individuals aged 60–80 years) who meet the inclusion criteria (either mild cognitive impairment or two or more cardiometabolic disorders) are being enrolled in this study at three different centers in Norway. Participants are block randomized to identically appearing capsules containing 80 mg of naturally purified anthocyanins or placebo 1:1. Dosage is 2 + 2 capsules per day for 24 weeks. The primary outcome will be the quality of episodic memory score, a composite measure from the extensively validated online cognitive test battery CogTrack®, which is administered at baseline and monthly for the next 6 months. Secondary outcomes include other major scores from CogTrack, as well as a range of neuroimaging and other biomarkers. Anthocyanin metabolites will be measured in blood and cerebrospinal fluid. The change from baseline scores will be subject to a mixed model for repeated measures analysis of covariance. The primary comparison will be the contrast (difference in the least-square means) between active and placebo at the end of the study (week 24). The primary study population will be a modified intention-to-treat population (ClinicalTrials.gov, NCT03419039).

**Discussion:** This study aims to demonstrate whether there are beneficial effects of purified anthocyanins on cognition and relevant biological functions in people at increased risk for dementia. Forthcoming results may contribute to further improvement of intervention strategies to prevent or delay the onset of dementia, including a potential decision to take anthocyanins toward phase III trials.

## Introduction

Dementia has become a rising public health problem, and the global socioeconomic burden of dementia is projected to amplify (1, 2). Misfolding of proteins such as amyloid, tau, and α-synuclein are key elements in the development of the most common neurodegenerative dementias such as Alzheimer's disease (AD) and Lewy body dementia. According to the most prominent hypothesis, the misfolding and aggregation of amyloid peptides are the key initial elements in AD (3). However, this is followed by secondary changes, including inflammation (4) and oxidative stress (5), which also contribute to neuronal dysfunction. Available treatments are only symptomatic, affecting cholinergic, and glutaminergic neurotransmission with modest clinical effect. There are no disease-modifying treatments available, and several phase III trials with anti-amyloid agents have failed (6) with some mixed but slightly more promising results with phase-III aducanumab recently reported (7, 8). Also, GV-971, a sodium oligomannate, from seaweed, has received conditional approval by China's National Medical Product Administration as a new oral treatment for mild to moderate AD (9). Notably, vascular risk factors, including diabetes, hypertension, and hypercholesterolemia, are associated with cerebrovascular disease, which is an important cause of a cognitive decline in older people and also associated with neurodegenerative diseases including AD (10). It is, therefore important to explore alternative disease mechanisms, such as inflammation (4), oxidative stress (5), and improvement of microcirculation as potential novel treatment targets.

Furthermore, dysregulation of cerebral capillaries, atherosclerosis, and endothelial changes also contribute to the development of AD (11). The strongest genetic risk factor for AD, the APOE e4 allele, is related to lipid metabolism (12), and diabetes and insulin resistance are established risk factors for AD (13). There are several studies (and reviews) that indicate a decrease in dementia incidence (14, 15). Although this in itself does not indicate that lifestyle can reduce dementia development, it is possible dementia risk can be reduced by lifestyle changes such as diet and physical activity (16).

Anthocyanins, a subclass of the flavonoids found in dark berries and fruits, are among the dietary factors that may have potentially positive effects on the pathogenesis of AD. Findings from cell, animal, and human studies suggest that they have antioxidant effects (17, 18), improve the blood lipid profile (19), and also have anti-inflammatory effects (20). Also, anthocyanins have been shown to increase flow-mediated dilatation (21–23) and to cross the blood–brain barrier (24). Thus, these substances have several effects relevant to protection against key mechanisms leading to cognitive decline and dementia in older people.

Interestingly, placebo-controlled studies have reported improvement of memory functioning in older people with memory problems or even dementia, after consumption of grape juice (25), blueberry juice (26), and cherry juice (27) as the source of anthocyanins. More recently, a randomized controlled trial has concluded that food-based anthocyanidin consumption was associated with a reduced risk of AD (28). However, previously published studies have major methodological limitations, including small sample sizes, short duration, and lack of biomarkers (29).

It is therefore not clear if these promising preliminary findings can be translated into clinically meaningful effects, that is, reducing the rate of cognitive decline and risk of dementia. Thus, they need to be substantiated in studies with a more robust design.

We have therefore set up a randomized, 24-week, parallel-group placebo-controlled three-center study: anthocyanins in people at risk for dementia.

The aim is to explore the potential of anthocyanin capsules to affect cognition in older people at risk for dementia favorably and also a range of relevant neuroimaging and peripheral biomarkers such as structural MRI, fluorodeoxyglucose positron emission tomography, and inflammation, oxidative stress, lipids, and other factors in the blood, cerebrospinal fluid (CSF), and gut microbiome. Also, we measure the effects on microcirculation and endothelial elasticity. Our recent pilot study showed that the anthocyanin capsules were well-tolerated (30).

## Methods

### Trial Design

This is a phase II, three-center, randomized, 24-week placebo-controlled trial enrolling 220 people living in three major cities in Norway (Stavanger, Oslo, and Bergen).

### Recruitment

Participants are being recruited from referrals to geriatric, psychiatric, neurology, cardiology, or memory outpatient clinics at two university hospitals and from advertisements in newspapers, radio, and on various social media. Furthermore, participants are recruited from the observational Dementia Disease Initiation study (31) and by actively reaching out to general practitioners in the areas.

### Trial Population Inclusion and Exclusion Criteria

#### Inclusion Criteria

People included in the study are those aged 60 years and older having an increased risk for dementia, by having either mild cognitive impairment (MCI) according to Winblad criteria (32) with or without cardiometabolic disorders (CMD), or normal cognition and ≥ 2 of the below stated CMDs known to be associated with increased risk of cognitive impairment and dementia (33–35): Stable cardiovascular disease, cerebrovascular disease, and metabolic disorders such as hypercholesterolemia, hypertension, or diabetes mellitus. The detailed inclusion criteria are displayed in Table 1.

**Table 1:** Principle inclusion and exclusion criteria for the trial.

**Inclusion criteria**	**Exclusion criteria**
Age ≥ 60 years and < 80 years and no, or on stable central nervous system medication for the past 3 monthsANDMCI according to Winblad criteria (32) *(i.e., meeting the general MCI following criteria: (a) the person is neither normal nor demented; (b) there is evidence of cognitive deterioration shown by either objectively measured decline over time and/or subjective report of decline by self and/or informant in conjunction with objective cognitive deficits; and (c) activities of daily living are preserved, and complex instrumental functions are either intact or minimally impaired)*ORhaving ≥ 2 of the following conditions known to be associated with increased risk of cognitive impairment and dementia: ➢Stable cardiovascular disease defined as coronary artery disease seen on angiogram ➢Cerebrovascular disease according to MRI criteria (i.e., presence of Fazekas score ≥2 points OR cerebral infarct (≥1 lesion) OR lacunar infarct (≥1 lesion) OR lobar microbleed (≥1 lesion), as judged by a qualified neuroradiologist) OR as visualized on CT scan for those having contraindications to MRI ➢Hypercholesterolemia, operationalized as a history of hypercholesterolemia and/or use of statin at baseline or serum cholesterol > 7 analyzed in screening blood test ➢Hypertension, operationalized as a previous diagnosis of arterial hypertension and/or use of antihypertensive drugs ➢Overweight (BMI > 25) ➢Diabetes mellitus type 1 ➢Diabetes mellitus type 2 (i.e., history of and/or use of oral antidiabetic drugs and/or HbA1c > 6.5%)	Any dementia (defined as CDR > 0.5) Other known relevant brain disease such as Parkinson's disease, normal pressure hydrocephalus, and other diseases which according to the study physician may cause cognitive decline Diagnosis of clinical stroke last 5 years Clinically significant depression, i.e., major depression or GDS-15 score ≥ 7 Unstable coronary heart disease Heart failure in need of treatment Systemic inflammatory diseases Other serious disease with expected survival < 5 years A somatic disease that might affect cognitive function adversely Usage of heparin, warfarin, Clopidogrel, or Ticagrelor or Non-Vitamin K antagonist Oral Anticoagulants Any use of Medox® during the 12 months prior to inclusion

*CAD, coronary artery disease; CDR, Clinician Dementia Rating scale; GDS, Geriatric Depression Scale; BMI, body mass index*.

#### Exclusion Criteria

The exclusion criteria are dementia; relevant brain disease such as Parkinson's disease; or other brain diseases (except AD or cerebrovascular disease), which according to the study physician (SP) may cause cognitive decline; clinically significant depression; a somatic disease that, according to the SP, might affect cognitive functioning adversely; usage of anticoagulants; and any use of the investigational product during the 12 months before inclusion. A full list of the exclusion criteria is also displayed in Table 1.

### Investigational Product

We use Medox® capsules, a standardized nutraceutical product that contains naturally purified anthocyanins from bilberry (*Vaccinium myrtillus*) and black currant (*Ribes nigrum*). The content of each capsule is as follows: 50% Maltodextrin Glucidex IT 19, 50% bilberry (*V. myrtillus*) and black currant (*R. nigrum*) extract powder with 80-mg anthocyanin citrates as the 3-O-rutinosides of cyanidin and delphinidin and the 3-O-b-galactopyranosides, 3-O-b-glucopyranosides, and 3-O-a-arabinopyranosides of cyanidin, peonidin, delphinidin, petunidin, and malvidin. Each capsule contains 80-mg anthocyanins, and the dosage chosen is four capsules, 320 mg/day, whereas the identically appearing placebo capsules contain 91% maltodextrin and 9% citric acid. This dosage has been reported to be associated with relevant biological alterations (19, 21, 30, 36) and to have good tolerability (30). The manufacturer of Medox®, MedPalett, is producing Medox® and identically appearing placebo capsules. The participants are instructed to take two capsules twice daily for 24 weeks, which are dispensed after randomization in the study. The anthocyanins and placebo capsules are identically packaged.

### Trial Procedures and Rationale

#### Screening Procedures

All potential participants are prescreened for eligibility by a telephone interview by study research assistants (SRAs) using a prescreening questionnaire. Information about the trial is mailed before the screening visit. Informed written consent is obtained before enrollment into the trial according to Good Clinical Practice principles. Participants are informed in the written informed consent forms that they have the right to withdraw from the study at any time without prejudice.

All eligible prescreened participants meet the SP and SRA for collection of relevant clinical data that include medical and psychiatric history, concomitant medications, both prescribed medications and over-the-counter medications, demographics, physical examination, and anthropometric measurements. Various blood panels are being collected, including blood count, metabolic panel, lipid panel, thyroid function tests, and blood tests related to vitamin B12 and folate serum level, and international normalized ratio for brief coagulation status assessment. These blood tests are being performed to exclude other medical illnesses, which may contribute to cognitive decline.

Cognition is assessed with the Mini-Mental State Exam, the Informant Questionnaire on Cognitive Decline in Elderly (37), and the Clinical Dementia Rating scale (38). The Geriatric Depression Scale (15 items) is also administered.

ECG, blood pressure, and pulse rate are recorded. Also, photoplethysmography, cardio–ankle vascular index, and flow-mediated dilation are performed in a subgroup.

Neuroimaging: Structural and functional brain MRI (alternatively CT scan if MRI is contraindicated) and fluorodeoxyglucose positron emission tomography imaging are performed in a subgroup of participants, dependent on the availability of relevant resources.

Venipuncture and lumbar puncture for collection, handling, and storage of blood and CSF are performed according to highly standardized operationalized procedures (see Supplementary Material). Fecal and urine samples for microbiome analyses are also collected.

Our main objectives for collecting neuroimaging and biological samples are to test whether relevant changes associated with dietary anthocyanin intake in participants with increased risk of progressive cognitive decline can be detected, for example, effects on the rate of loss of brain structure, cortical metabolic activity, and on oxidative and inflammation markers in the blood, CSF, and the microbiome.

Second, we will investigate whether any biomarker changes are associated with the various cognitive domains assessed with the cognitive tests.

We will also perform subgroup analyses to explore whether people with a CSF and/or MRI pattern typical of AD or cerebrovascular disease respond differently to anthocyanins compared with those without such changes. These biomarker analyses will be tentative, as the study is not powered for subgroup analyses but may be informative for planning a potential phase III trial.

#### Baseline Assessment

At the baseline visit, any unplanned changes in medications since the screening assessment are recorded, and any future changes are discouraged if safe.

Participants are asked to maintain their lifestyle and habitual diet during the intervention.

The capsules are then distributed to participants after randomization (see later).

The participants will be trained in performing the CogTrack® System, an online set of cognitive tests with proven utility, reliability, sensitivity, and validity (39), as well as reliable sensitivity to change over time (40); (see later). The battery of tests includes previously described tests, such as simple reaction time, choice reaction time, digit vigilance, immediate and delayed word recall and recognition, pattern separation, spatial working memory, and numeric working memory (41). The baseline test will be performed at home on two occasions before taking the first study dose and then monthly for 6 months. Collection and registration of the cognitive data will be performed securely online (39). Cognitive data are stored on secure Amazon EC2 cloud servers with encryption in transit (transport layer security) for data across services. Data will be uploaded to Google Drive, into a folder where only authorized persons from Wesnes Cognition can access using two-step verification. Once the download is complete and confirmed by the study team as received, the data will be removed from the shared folder. A copy of the data remains on the Amazon EC2 databases. Participants are instructed to perform the test at the same time of day on all occasions, usually in the morning, and to ensure testing procedures are standardized as much as possible (i.e., temperature, room, research associate, and coffee intake).

#### Randomization, Allocation and Blinding

Participants are randomized to anthocyanins or placebo in a 1:1 ratio based on stratified block randomization. The strata are made up of the combination of three treatment sites [Stavanger University Hospital [SUS], Akershus University Hospital [Ahus], Oslo, and Betanien Hospital, Bergen] and two recruitment groups, that is, those who are recruited due to CMD criteria only vs. those who are recruited due to cognitive criteria with or without CMD criteria. Within each of the six strata, the randomization is performed in blocks of varying sizes (four or six) to ensure local balance and non-predictability of allocation. The randomization lists with ID numbers and anonymous treatment arms (“A” and “B”) are produced by the study statistician and sent to the production site, which is instructed to do a random allocation (using a dice) to specify which of A and B should be the intervention arm. The capsules (anthocyanins and placebo) are produced according to the randomization lists, marked with ID numbers, and distributed to the participating centers. At the centers, the participants are numbered consecutively at their inclusion in the study. All study staff, participants, and the data analyst remain blinded to the actual treatment given until data have been analyzed.

#### Follow-Up Assessments

**Figure 1:**
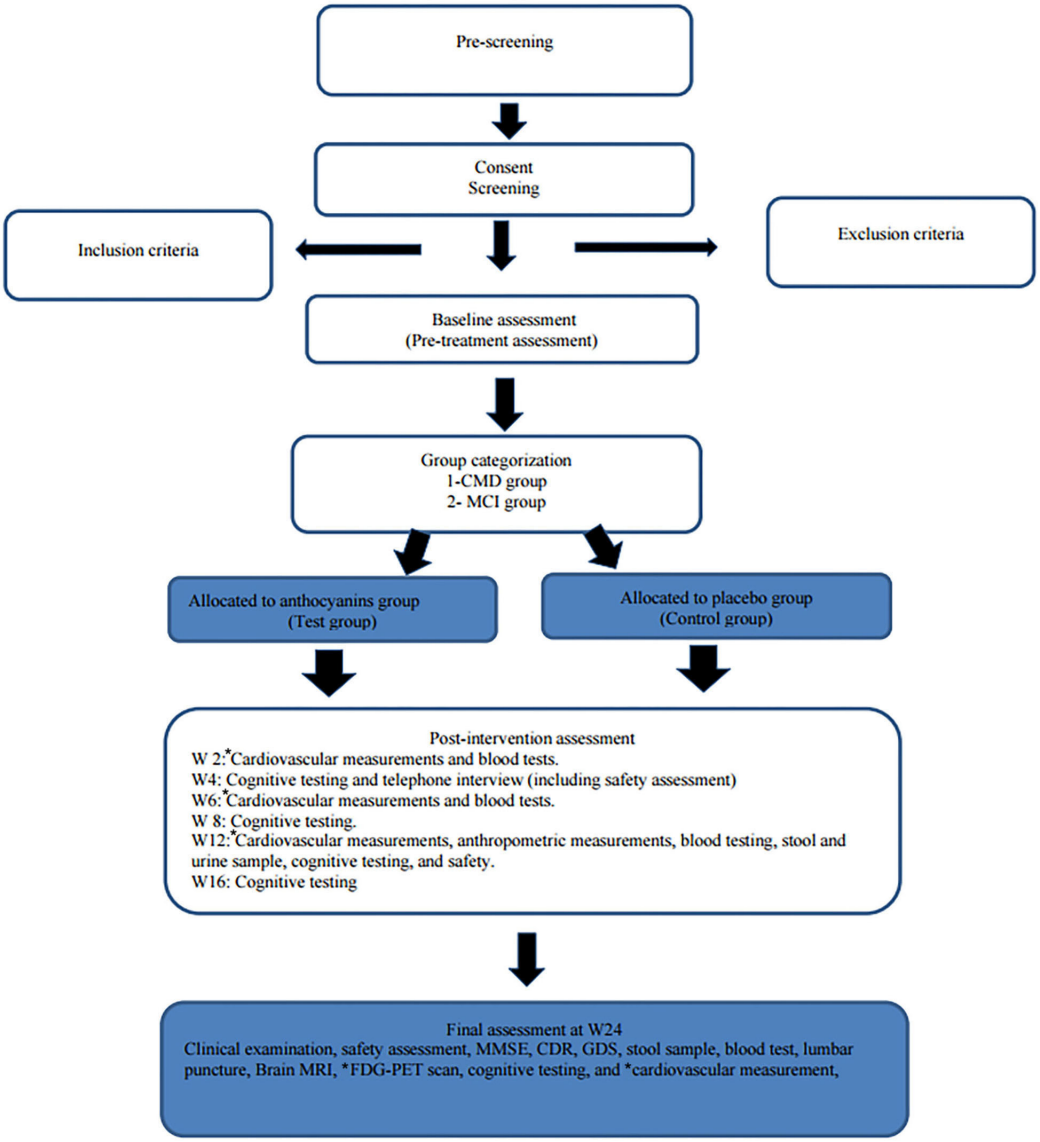
Flowchart of the trial. SUS, Stavanger University Hospital; Ahus, Akershus University Hospital; BH, Betanien Hospital, CMD, cardiometabolic disorders; MCI, mild cognitive impairment; FDG-PET, fluorodeoxyglucose (FDG) positron emission tomography (PET); CAVI, cardio–ankle vascular index; FMD, flow-mediated dilation; PPG, photoplethysmography; MMSE, Mini-Mental State Examination; CDR, Clinician Dementia Rating scale; IQCODE, The Informant Questionnaire on Cognitive Decline in the Elderly; GDS, Geriatric Depression Scale. *cardiovascular measurements are only performed at SUS and Ahus. *FDG-PET scan is only performed at SUS.

Participants are contacted by telephone after 4 weeks (for discussion of safety and procedures) and seen in the clinic at weeks 12 and 24 (final visit). Blood tests are also being collected at weeks 12 and 24 (and weeks 2 and 6 for those performing cardiovascular measurements). Lumbar puncture is performed at baseline and again at week 24, and cardiovascular measurements are performed at weeks 2, 6, 12, and 24. Feces and urine sampling are collected at weeks 12 and 24. All the clinical visits are recorded in the medical journal and the case report form. See the study clinical assessment summary in Table 2 and the study flowchart in Figure 1.

**Table 2:** Schedule of enrollment, intervention, and follow-up of the trial.

	**Screening** **W6–0**	**Baseline** **W0**	**W2**	**W4**	**W6**	**W8**	**W12**	**W16**	**W20**	**W24**
− Informed consent − Eligibility	x									
− Randomization		x								
− Dispense trial capsules		x					x			
− Concomitant medications	x	x		x			x			x
− Heart rate − blood pressure	x		x		x		x			x
− Height − Weight − BMI	x						x			x
− ECG	x		x		x		x			x
− Neuroimaging − (MRI, FDG-PET)	x									x
− Cardiovascular measurements	x		x		x		x			x
− Blood sampling	x		x		x		x			x
− Lumbar puncture	x									x
− MMSE − CDR − GDS	x									x
− IQCODE	x									
− Stool sample − Urine sample	x						x			x
− CogTrack training	X									
− CogTrack (monthly)		x		x		x	x	x	x	x
− Telephone interview (compliance, side effects)				x						
− Compliance check (Collection of bottles/blisters)							x			x
− Adverse event monitoring				x			x			x
− Question about change in lifestyle				x			x			x

*BMI, body mass index; ECG, electrocardiogram; FDG-PET, fluorodeoxyglucose positron emission tomography; CAVI, cardio–ankle vascular index; FMD, flow-mediated dilation; PPG, photoplethysmography; MMSE, Mini-Mental State Examination; CDR, Clinician Dementia Rating scale; IQCODE, The Informant Questionnaire on Cognitive Decline in the Elderly; GDS, Geriatric Depression Scale*.

Upon the beginning of the study, a simplified version of Table 1 is distributed to the participants to enable them to follow the study phases.

#### Trial Medication Adherence and Monitoring

To be able to calculate medication adherence, the participants will be instructed to write the time and date of first and last capsule intake on the medicine package and to collect and return the remaining capsules. These need to be brought along at the next follow-up. The following formula will be used to calculate medication adherence (42):

$$\begin{array}{l} \frac{Number\;of\;capsules\;taken}{Number\;of\;capsules\;the\;participant\;should\;have\;been\;taken}x100=n\;\% \end{array}$$

The number of capsules the participant should have taken will be calculated based on the number of days between the first and last capsule intake. The remaining capsules in the package will be subtracted from the total number of capsules to yield the number of capsules taken. For example, if the participant should have taken 360 capsules but have taken 324 capsules, the medication adherence will be (324/360) × 100 = 90%. Medication adherence will be calculated between 0 and 12 weeks and from 12 to 24 weeks. Also, in the telephone interview, after 4 weeks, the intake of capsules per protocol and any adverse effects will be noted. Anthocyanin metabolites will also be measured in blood, CSF, and urine.

### Outcome Measures

#### Primary Outcome Measure

Cognitive assessment: CogTrack® is an online cognitive test battery consisting of 10 subtests, which, based on factor analysis, are combined into the following domains: attention, memory, and cognitive speed. There are 14 different word lists, each with 15 words, for the verbal memory test. The instructions are presented visually at the start of each testing session and also at the start of each task. In-task responses are made using the right arrow on the keyboard in two tasks and the left and right arrows in the other two. The participants are instructed to rest their finger(s) lightly upon the key(s) throughout each task. The speed and accuracy of every response are recorded. The primary outcome measure will be the quality of episodic memory combination of two accuracy scores from each of immediate and delayed word recall, word and picture recognition (four tasks in total), which has been shown to represent the overall quality of episodic memory and to be sensitive to cognitive changes, with negligible learning effects (39, 40). The battery is completed online monthly at home, using different word-lists at each occasion.

#### Secondary Outcome Measures

See test detail outlines in (39).

1. Secondary endpoints from the CogTrack System will include:
  - attentional intensity index
  - sustained attention index
  - cognitive reaction time
  - attentional fluctuation index
  - quality of working memory
  - speed of memory retrieval.

### Safety and Handling of Adverse Events

Anthocyanins are safe (30), and there are no serious adverse effects to be expected. The study will nevertheless focus on safety, as explained later.

#### Recording Adverse Events

At each study visit, the participants are proactively asked about the occurrence of any adverse experiences, particularly about any abnormal bleeding since his/her last visit. The site SRA and SP will also assess adverse events by assessment of clinical and laboratory features. All adverse events, whether observed by the SP or elicited from or volunteered by the participant, will be documented. Blood is analyzed for safety with routine clinical analyses (liver, kidney, hematology, and international normalized ratio) at weeks 12 and 24. The electrocardiogram is taken at study inclusion and at week 24. Telephone and e-mail addresses of the SRA and SP are available to facilitate easy communication about possible adverse events. If dementia or other new medical conditions are detected during the screening process, this information is forwarded to the general practitioner if the participant agrees. If deemed necessary, action may also be taken by the SP.

#### Serious Adverse Events

Any serious adverse events during the study are recorded, and actions from the SRA and SP are taken as needed. The chief investigator (CI) is to be notified within 24 h of investigator awareness of the event.

### Emergency Unblinding

Given the safety profile of anthocyanins that have been reported (30, 43), we expect the need for emergency unblinding to be extremely rare. Nevertheless, we have the following procedure in case emergency unblinding is required:

If unblinding is deemed to be necessary for the event of significant concerns regarding participant safety, the CI will be notified immediately by the SP. If the CI considers emergency unblinding necessary, a request will be directed to appointed personnel at the MedPalett. The requested information will be transmitted to the requesting party. The CI will coordinate the process.

The actual allocation must not be disclosed to the participant or other trial personnel since the trial is placebo controlled-double-blind. Emergency unblinding should not necessarily be a reason for trial drug discontinuation. Details of any emergency unblinding shall be well-documented.

### Governance and Monitoring

The sponsor of the study is SUS. The clinical research group includes Ph.D. candidates, postdocs, and clinicians from the three different centers. A trial manager is in day-to-day charge of the trial and responsible for trial documentation, supporting the centers to recruit promptly, monitoring compliance with the protocol, and organizing meetings of the trial management group. This group includes CI, the local principal investigators, an external expert, and the trial manager. A decision-making group, with members from the clinical research group, will discuss relevant medical or ethical issues via e-mail.

### Data Storage

All parties will ensure the protection of subject personal data, and subject names or other identifiable data are not included in any reports, publications, or other disclosures, except where required by law. Data are stored securely, both on paper, on password-protected computers at the site, and encrypted online, at SUS with locked doors and shelves, with access only to dedicated study personnel.

### Statistical Considerations

#### Sample Size Calculations

The sample size calculations are based on published data on CogTrack (40). We will assume a Cohen's d effect size of 0.4, two-tailed testing at *p* < 0.05, at 80% power, which is considered to be a clinically relevant effect. A sample size of 110 patients per arm is required, allowing for ~10% dropout. Analyses will be adjusted for baseline scores for each test. *A priori* subgroup analyses are planned, including separate analyses of those with MCI vs. normal cognition, old vs. middle-aged (defined as above or below median age), participants with and without CMD, and those with normal and those with abnormal CSF AD markers, although the study is not powered for these analyses.

#### Planned Statistical Analyses

The primary analysis will be the comparison between the active and placebo groups on CogTrack quality of memory domain score change during follow-up. The primary study population will be a modified intention-to-treat population, that is, all participants having ingested at least one capsule and with at least one follow-up assessment.

Summary statistics (n, mean, standard deviation, and minimum and maximum scores) will be calculated by treatment arm for the data at each testing session, and, additionally, for the change from baseline scores. A mixed model for repeated measures ANCOVA will analyze the change from baseline scores. The primary comparison will be the contrast (difference in least-square means) between active and placebo at the end of the study (week 24) (44). A detailed statistical analysis plan will be finalized before the unblinding of data.

#### Trial Status

The study started the recruitment of participants in April 2018. We are expecting to reach the recruitment target in summer 2020 and expect completion of the study during 2020.

## Discussion

Out of 236 participants who have been assessed for eligibility, 50 participants were excluded (49 not meeting the inclusion criteria, and one declined to participate). As of the end of February 2020, 181 participants have been randomized; 123 in the CMD group and 58 in the MCI group. Among the strengths of this trial is the relatively large sample size, making it one of the largest anthocyanin trials and sufficiently powered to detect a medium effect of anthocyanins on primary cognitive outcome measures. The use of web-based cognitive assessment enables frequent testing without learning effects, which is increasing the statistical power. The comprehensive acquisition of biomarkers allows for a detailed mechanistic study of anthocyanin effects. Finally, the collection of blood and CSF for anthocyanin metabolite analyses allows for analysis of dose effects. However, some limitations of this trial are the lack of dietary records and not giving specific dietary instructions concerning the intake of anthocyanin-rich foods. However, participants are asked to maintain their habitual diet during the intervention period. Of note, applying any dietary restrictions in a long-term trial lasting 24 weeks might challenge participant's compliance. Importantly, the study has a washout period of 12 months after any Medox® intake to provide a period of abstinence from the interventional product.

The 6 months' trial duration is similar to previous trials with cholinesterase inhibitors and expected to be sufficiently long to detect differences in terms of symptoms but might be too short to detect disease-modifying effects on the risk to progress to dementia or biology. The number of participants with MCI is lower than the group with CMD, resulting in reduced power for sub-analysis in this group. The participants are largely ethnic Norwegian, that is, Caucasians, with relatively high socioeconomic status and educational level. Thus, the findings might not be immediately relevant to other populations. Some initial characteristics based on the first 86 randomized participants at SUS (pre-intervention) are displayed in Table 3. Finally, anthocyanins might have biological effects, for example, stool color change or constipation, with the risk for participants or research staff to become unblinded. However, there might be many possible explanations for any such changes unrelated to the study.

**Table 3:** Some initial baseline characteristics based on the first 86 randomized participants at SUS.

**Variables**	**Both groups (*n* = 86)**	**CMD group (*n* = 60)**	**MCI group (*n* = 26)**
Men, n (%)	47 (55%)	33 (55%)	14 (54%)
Age in years	68 (65, 74)	68 (64, 74)	69 (66, 76)
Education in years	14 (11, 16)	14 (11, 16)	14 (12, 17)
MMSE score	29 (28, 30)	30 (28, 30)	29 (27, 30)
No. of concomitant medications	3 (2, 5)	3 (2, 5)	2 (1, 4)

*Data are presented as median (interquartile range, IQR) unless otherwise specified*.

*SUS, Stavanger University Hospital; CMD, Cardiometabolic disorders; MCI, mild cognitive impairment; MMSE, Mini-Mental Status Examination*.

## Ethics Statement

The studies involving human participants were reviewed and approved by The Norwegian regional ethics committee (2017/374). The patients/participants provided their written informed consent to participate in this study. The trial has been registered at: http://www.clinicaltrials.gov/, NCT03419039.

## Service User Involvement

The valuable feedback provided by our participants in our pilot study (30) was taken into consideration in developing the anthocyanins in people at risk for dementia study design. The user involvement representatives at the center for age-related medicine at SUS have contributed by giving their inputs and recommendations to develop the advertising flyers and have also helped to promote the publicity of the study and recruitment. Also, there is a plan for user involvement also in interpretation, dissemination, and presentation of the study outcome.

## Author Contributions

DA, HS, and AB devised the main conceptual idea and developed the design of the study, drafted the original protocol, and worked out the ethical committee and clinical trial registration procedures. CB contributed to the design of the protocol. KO defined the neuroimaging protocol. AL defined the cardiovascular measurements. HS, AB, and KK are conducting the study and collecting the data. KW planned the cognitive test battery and will perform the CogTrack analyses. KW and ID defined the statistical analyses with input from DA. KK wrote the manuscript with input from AB, DA, HS, KO, RO, CB, ID, KW, and HB. All authors contributed to manuscript revision, read, and approved the final manuscript.

## Conflict of Interest

AB has received support for conference participation from Evonik. DA has received research support and/or honoraria from Eisai (Evonik), Biogen, NSC Therapeutics, and GE Health. This paper represents independent research partly funded by the National Institute for Health Research Biomedical Research Centre at South London and Maudsley National Health Service Foundation Trust and King's College London. The views expressed are those of the author and not necessarily those of the National Health Service, the National Institute for Health Research, or the Department of Health and Social Care. KW owns 100% of Wesnes Cognition Ltd., which developed and holds the worldwide copyright and Trademark for CogTrack^®^. His company will receive financial compensation to reflect the per-use fee and other overheads for the use of the System in the study, as well as for his assistance in interpretation, access to the CogTrack database, and the conduct of the statistical analysis of the data from the System. HB was employed by company Ecog Pro Ltd., Bristol, United Kingdom. The remaining authors declare that the research was conducted in the absence of any commercial or financial relationships that could be construed as a potential conflict of interest. The reviewer GS declared a past co-authorship with several of the authors TF, DA, HS, RO, and CB to the handling editor.

## Abbreviations

AD: Alzheimer's disease
APOE e4: Apolipoprotein E e4
MCI: mild cognitive impairment
CMD: cardiometabolic disorders
SP: study physician
SRA: study research assistant
SUS: Stavanger University Hospital
Ahus: Akershus University Hospital
CI: chief investigator.
