# Supplementary material for: Effects of Purified Anthocyanins in People at Risk for Dementia: Study Protocol for a Phase II Randomized Controlled Trial
Source: Front Neurol. 2020 Sep 2;11:916. doi: 10.3389/fneur.2020.00916 (PMC7492209; doi:10.3389/fneur.2020.00916)
Supplement: Supplementary file 1 [file Data_Sheet_1.PDF]

frontiers

Effects of Purified AnthoCyanins in People at Risk for Dementia (ACID): Study Protocol for

a Randomised Controlled Trial

1 Supplementary Data

1.1 Biomarkers acquisition in ACID study

1.1.1 Stool samples:

1.1.1.1 Sample collection:

Feaces samples are collected from participants at baseline, W12 and W24. The samples are sent by the participants to Evonik site Halle-Künsebeck– Germany via prepaid shipping label provided by study site.

1.1.1.2 Handling and storage of stool samples:

Arrival of samples at Evonik site Halle-Künsebeck at room/ambient temperature.

Content of the collection tubes is divided into 3 aliquots and stored at -80 °C

Access to freezer and sample information is restricted and controlled.

After completion of the study, the samples will be processed: DNA isolation and sequencing of the 16S rRNA gene sequences

1.1.1.3 Sample labelling:

From each collection tube 3 times 1,5 ml of the sample is transferred into 2 ml tubes, and then 2 ml tubes are labelled in the following manner:
'P-XXX_T-YY_Z' e.g. P-055_T-12_A, P-055_T-12_B and P-055_T-12_C
P = Proband / test person
XXX: number of test person
T = Time
YY: Time (weeks; 0, 12 or 24)
Z: number of aliquot (tube A, B, C) ☾ Storage in racks at -80°C
Collection tubes (originally labelled) are stored at -80°C

1.1.2 Urine samples:

1.1.2.1 Sample collection:

An early morning urine sample is collected at study site at baseline, W12 and W24.

1.1.2.2 Handling and storage of urine samples:

Samples are pipetted into 3 aliquots, labelled, and then stored at -70°C.

1.1.3 Peripheral blood samples:

1.1.3.1 Sample collection:

Blood tests are collected at week 2, 6 12 and 24.

1.1.3.2 Handling and storage of samples:

EDTA 1 x 6ml for anthocyanins, then the following steps:

- Put in ice / ice water

- Centrifuge at 4 ° C 1800G for 15min

- Transfer supernatant to new primary test tube.

- Mix gently while shaking the test tube

- Distribution: 2 aliquots of 600µL + 12µL formic acid

- Green cork

- Place in - 75 ° C freezer in labeled box "Plasma Antocyanins "with visit number written on it".

The test tube is labeled with a label marked "CORROSIVE: Added 12µL formic acid.

Will be stored in refrigerator for sample distribution. Spinned in centrifuge 2-3min before sample distribution (due to evaporation to cork during storage).

EDTA 1 x 6ml for cytokines, then the following steps:

- Will put it on ice / ice water

- Centrifuge at 4 ° C 2400G for 15min

- Put aliquot test tubes on cold board/tray (ice)

- Distribution: 4 aliquots of 500µL (minimum 300µL / aliquot)

- Use label "EDTA plasma cytokines"

- Place in - 75 ° C freezer in box labeled "EDTA plasma cytokines.

EDTA 2 x 6ml, then the following steps:

- Centrifuged at 1200G for 15 minutes
- Distribution: to 8-10 aliquots of 600µL
- Purple cork
- Use label labeled "EDTA plasma"
- Place in - 75 ° C freezer in box labeled "EDTA plasma "with visit number written on it".

EDTA 1 x 6ml complete blood count, then the following steps:

- Distribution: to 5 aliquots of 900 µL
- Blue cork
- Use label labeled " complete blood count"
- Insert - 75 ° C freezer in box marked with " complete blood count"

Serum gel 4 x 5ml, then the following steps:

- Centrifuge at 1200G for 15 minutes
- Distributed to 8 aliquots of 900µL
- Red cork
- Label it with "serum" label.
- Place in - 75 ° C freezer in box labeled "serum- with visit number written on it".

1.1.4 CSF:

1.1.4.1 Sample collection:

Lumbar puncture is performed at baseline and week 24.

1.1.4.2 Handling and storage of samples:

We will collect 4 test tubes of spinal fluid. Must be handled within 1 hour.

- Tube 5ml (red cork) 1 x 2ml for anthocyanins → put on ice / ice water

- Tube 5ml (red cork) 2 x 4ml for distribution to CSF aliquots → room temperature.

- Nunc tube 4.5ml (or 3.6ml) 1 x about 3ml spinal fluid for dementia markers → room temperature

Spinal fluid for anthocyanins → Bio-branches tubes with 2ml spinal fluid on ice, then the following steps:

- Centrifuge at 4 ° C 1800G for 15min

- Take from supernatant to new primary tube

- Mix gently while shaking the tube (4-5 times)

- Use pipette with polypropylene tip

- Distributed into 2 aliquots of 600µL + 12µL formic acid

- Use label labelled with "CSF anthocyanins"

- Place in - 75 ° C freezer in box labelled "CSF antocyanin visit number

The tube is labelled with a label marked "CORROSIVE: Contains 12µL formic acid".

Store in refrigerator for sample distribution. Spin in centrifuge 2-3min before sample distribution (due to evaporation to cork during storage)

Spinal fluid 2 glasses of 4ml, then the following steps:

- Centrifuge at room temperature 2000G for 10min

- Transfer supernatant to new primary tube.

- Mix gently while turning the tube (4-5 times) distribution:

- 14 x 450 µL aliquots → Green cork

  Label: CSF, marked business and date

Place in - 75 ° C freezer in box labelled with: "visit number"

Spinal fluid Nunc tube 4.5ml, then the following steps:

- Centrifuge at room temperature 2000G for 10min

- Transfer supernatant to new primary tube.

- Mix gently while turning the tube (4-5 times) distribution:

- Use pipette with polypropylene tip

- 2x 1ml (or more) spinal fluid for 2x 3.6ml NUNC tubes →

Label: dementia markers

Place in - 75 ° C freezer in box labelled: dementia markers with “visit number”
